# Seroprevalence of SARS-CoV-2 and humoral immune responses to COVID-19 mRNA vaccines among people who use drugs - in the light of tailored mitigating strategies

**DOI:** 10.1186/s12954-024-01023-9

**Published:** 2024-06-19

**Authors:** Linda Elise Couëssurel Wüsthoff, Fridtjof Lund-Johansen, Kathleen Henriksen, Gull Wildendahl, Jon-Aksel Jacobsen, Leni Gomes, Hina Sarwar Anjum, Regine Barlinn, Anne-Marte Bakken Kran, Ludvig Andre Munthe, John T. Vaage

**Affiliations:** 1 Unit for Clinical Research on Addictions, Oslo University Hospital, PO Box 4959 Nydalen, Oslo, 0424 Norway; 2 Norwegian Centre for Addiction Reasearch, Institute of Clinical Medicine, University of Oslo, PO Box 1039 Blindern, Oslo, 0315 Norway; 3 Department of Immunology, Oslo University Hospital, PO Box 4950 Nydalen, Oslo, 0424 Norway; 4 ImmunoLingo Convergence Center, Institute of Clinical Medicine, University of Oslo, Gaustadalleen 21, Oslo, 0349 Norway; 5 Institute of Clinical Medicine, University of Oslo, PO Box 1171 Blindern, Oslo, 0318 Norway; 6 Agency for Social and Welfare Services, Oslo Municipality, PO Box 30 Sentrum, Oslo, 0101 Norway; 7 Student Health Services, Student Welfare Services in Oslo, Problemveien 9, Oslo, 0313 Norway; 8 Department of Microbiology, Oslo University Hospital, PO Box 4950 Nydalen, Oslo, 0424 Norway; 9 Division of Infection Control, Norwegian Institute of Public Health, PO Box 222 Skøyen, Oslo, 0213 Norway; 10 KG Jebsen Centre for B cell Malignancies, and Precision Immunotherapy Alliance, Institute of Clinical Medicine, University of Oslo, PO Box 4950 Nydalen, Oslo, 0424 Norway; 11 Precision Immunotherapy Alliance, Institute of Clinical Medicine, University of Oslo, PO Box 1171 Blindern, 0318 Oslo, Norway

**Keywords:** SARS-CoV-2, COVID-19, Substance use disorders, Seroprevalence, Antibody response, Corona vaccine, People who use drugs

## Abstract

**Background:**

During the initial wave of the COVID-19 pandemic, there was a surprisingly low incidence of SARS-CoV-2 among People Who Use Drugs (PWUD) in Oslo, Norway, despite their heightened vulnerability regarding risk of infection and severe courses of the disease.This study aims to investigate the seroprevalence of SARS-CoV-2 antibodies among PWUD, their antibody responses to relevant virus infections and COVID-19 mRNA vaccines, and their vaccination coverage compared to the general population.

**Methods:**

Conducted as a prospective cohort study, data was collected from residents in six institutions for homeless PWUD and users of a low-threshold clinic for opioid agonist treatment. Ninety-seven participants were recruited for SARS-CoV-2 seroprevalence analysis. Additional two participants with known positive SARS-CoV-2 test results were recruited for further analyses. Twenty-five participants completed follow-up. Data included questionnaires, nasal swabs and blood samples. Data on vaccination coverage was obtained from the National Vaccine Register. Serologic methods included detection of antibodies to relevant virus proteins, neutralizing antibodies to SARS-CoV-2, antibodies to the full-length spike protein, and receptor-binding domain from SARS-CoV-2.

**Results:**

Among PWUD, antibodies to SARS-CoV-2 were detected in 2 out of 97 samples before vaccines against SARS-CoV-2 were available, comparable to a 2.8% frequency in population-based screening. Levels of serum antibodies to seasonal coronaviruses and Epstein-Barr-Virus (EBV) in PWUD were similar to population-based levels. After the second vaccine dose, binding and neutralizing antibody levels to SARS-CoV-2 in PWUD were comparable to controls. Eighty-four of PWUD received at least one dose of COVID-19 mRNA vaccine, compared to 89% in the general population.

**Conclusion:**

Results indicate that PWUD did not exhibit increased SARS-CoV-2 seroprevalence or elevated serum antibodies to seasonal coronaviruses and EBV. Moreover, vaccine responses in PWUD were comparable to controls, suggesting that vaccination is effective in conferring protection against SARS-CoV-2 also in this population.

## Background

The World Health Organization (WHO) was first alerted about several cases of viral pneumonia in Wuhan, China, on December 31st 2019. On January 9th 2020 it was evident that the outbreak was caused by a novel coronavirus, later called severe acute respiratory syndrome-related coronavirus (SARS-CoV-2) and the disease caused by this virus called coronavirus disease 2019 (COVID-19). The first confirmed cases in the European region came February 24th in France, and on March 11th 2020, WHO characterized COVID − 19 as a pandemic [1, 2].

The first SARS-CoV-2 case in Norway was confirmed on February 26th, 2020 [3]. The knowledge about this virus was scarce at the time. Early experiences from the Wuhan outbreak underscored high mortality rates, rapid disease progression, and heightened risk among older individuals and those with chronic conditions [4–8]. The strain on healthcare systems was palpable [6, 9]. To mitigate these scenarios, Norway implemented extensive measures, including a nationwide lockdown from March 13, 2020 [10]. This involved the closure of all non-emergency services. A gradual reopening of society began on April 7, 2020, starting with kindergartens and schools. This process continued until August when new restrictions were imposed in response to the evolving pandemic situation [11, 12].

Already from the beginning of the pandemic, great concern was raised in regard to the spread of SARS-CoV-2, and the severity and mortality related to COVID-19 among people who use drugs (PWUD) [13–21]. This concern has later been supported by several studies finding that PWUD are at greater risk of being diagnosed with COVID-19, having a more severe course of the disease and also greater mortality related to the disease [22–25].

Oslo has about 700 000 inhabitants with an estimated 1556 (CI95% 1236–1880) people who inject drugs (PWID) in 2021 [26]. The Agency for Social and Welfare Services in Oslo municipality (ASWS) offers health and social services to PWUD 18 years and older, including housing facilities (institutions), low-threshold health services, needle exchange services, and a supervised drug consumption site. In 2021, 1206 people were living in one of the ASWS’s institutions for PWUD [27]. Comprehensive community services are provided in close collaboration between the ASWS, non-governmental organizations (NGOs) and user organizations. The specialist health services provide substance use treatment including opioid agonist treatment (OAT). In addition, a low-threshold clinic for OAT is operated in collaboration between the specialist health service and the ASWS [28].

### Risk management

The emergency management at the ASWS implemented extensive measures to prevent and handle the risks of spread of SARS-CoV-2 among PWUD. These included frequent indoor and outdoor testing, routines for quarantine at the institutions, and performing risk evaluations and developing emergency plans for each resident. Further, separate institutions for quarantine (for people with risk of infection without verified positive SARS-CoV-2 test result) and isolation (for people with verified positive SARS-CoV-2 test result) were established for PWUD. These institutions included substance use treatment to aid withdrawal symptoms, as well as a contingency plan in the event that emergency services and hospitals became overloaded. Ambulatory services (including delivery of OAT) were established in close collaboration between the ASWS, NGOs, speciality health services and user organizations to compensate for closed low-threshold in-door services during the “lock-down”. Health personnel in the ASWS were reorganized to aid in the above-mentioned measures, and nurses and doctors voluntarily engaged in working 24/7-on-call shifts. Additionally, information materials about SARS-CoV-2, preventative measures, symptoms, testing and when and how to seek help from health services were produced in close collaboration with user organizations and disseminated through handouts and social media. The vaccines against SARS-CoV2 became available during the first months of 2021. PWUD were prioritized for vaccination right after elderly and people with chronic diseases from April 2021.

The emergency management at the ASWS was preparing for an overwhelming number of infected and severely ill persons among PWUD. However, during the first wave of the pandemic very few PWUD tested positive or had severe courses of the disease. We hypothesized that there had not been a spread of SARS-CoV-2 among PWUD, that PWUD had asymptomatic courses of COVID-19 and, thus, had not been tested, or that PWUD had acquired immunity towards SARS-CoV-2 through previous virus infections. Furthermore, as the vaccines against SARS-CoV-2 were novel and little was known about antibody responses and vaccine acceptance among PWUD, we aimed to investigate the antibody response to vaccines against SARS-CoV-2 and vaccination coverage among PWUD compared to the general population.

### Aims

The aims of this study were to investigate the seroprevalence of SARS-CoV-2, the antibody responses to seasonal coronaviruses and EBV and to vaccines against SARS-CoV-2, together with the vaccination coverage in PWUD compared to the general population.

## Materials and methods

### Design

This was a prospective cohort study.

### Setting

The data was collected from residents at six different institutions in the ASWS and users of the low-threshold clinic for OAT.

The institutions of the ASWS are low threshold housings with bed-sitting rooms for homeless PWUD from 18 years and above, many of which have co-morbid somatic and psychiatric illnesses. The institutions are staffed with social workers and some also have health personnel.

### Participants

Residents at institutions of the ASWS where informed about the study by staff members and recruited by health personnel. All participants were invited to take part in both the present study and in the broader COVID-19-study which encompassed all patients at Oslo University Hospital and all blood samples analyzed for a large biobank. The latter study also facilitated the collection of data through registries, such as the National Vaccine Registry. All, but one, consented to participate in both studies.

The inclusion criteria were being a resident at one of the ASWS’ institutions for PWUD or being a patient at the low-threshold clinic for OAT, and consenting to participate in the study. The exclusion criteria were not being able to consent, i.e. because of psychosis or heavy drug intoxication, or being under the age of 18. Two of the residents were asked to participate on basis of known positive SARS-CoV-2-PCR test results. One of them had an ongoing SARS-CoV-2 infection at the time of inclusion, the other had tested positive one month earlier. These two participants were not included in the seroprevalence analyses. The second data collection was planned to take place 3 months after the participants had received their second dose of vaccine against SARS-CoV-2. Information on vaccination coverage was collected from those that consented also to participate in the COVID-19 study.

Three reference groups were used for the analyses of SARS-CoV-2 seroprevalence. To compare results with the general population, blood samples were obtained from the COVID-19 biobank at Oslo University Hospital. The biobank contains sera from healthy volunteers that were obtained from participants in the Norwegian Coronavirus study, the Mother and Child study, the NorFlu study and health care workers. These samples were divided into a prepandemic sample (i.e. collected in 2019, *n* = 1728), a COVID-19 convalescent sample (*n* = 433), and a healthy control sample (i.e. not previously tested postitive for SARS-CoV-2) (*n* = 648). The two latter control samples were collected in an overlapping period with those from the PWUD donors (i.e. Nov-Dec 2020).The healthy control sample was also used as reference for the analyses of antibodies to seasonal coronaviruses and Epstein-Barr virus. The “prepandemic” and “COVID-19 convalescent” samples were chosen as reference groups to provide specificity and sensitivity of the SARS-CoV-2 antibody analyses. The “healthy control” sample was chosen as reference group to compare seroprevalence in the study population to the general population.

Two reference groups were used for the analyses of antibodies against COVID-19 mRNA vaccines. As reference for immunocompromised individuals, we used published data from a cohort of vaccinated patients treated with anti-CD20 antibodies for Multiple Sclerosis [29] (*n* = 337). The other reference group consisted of samples from healthy vaccinated health care workers, the Norwegian Coronavirus study and blood donors from the COVID-19 biobank described above (*n* = 267). These reference groups were chosen to compare the antibody responses to COVID-19 mRNA vaccines in the study population with a group of immunocompromised individuals and the general population.

The vaccination coverage in the study population was compared to the vaccination coverage in the corresponding age group of the general population using data from the National Vaccine Registry [30].

**Fig. 1:**
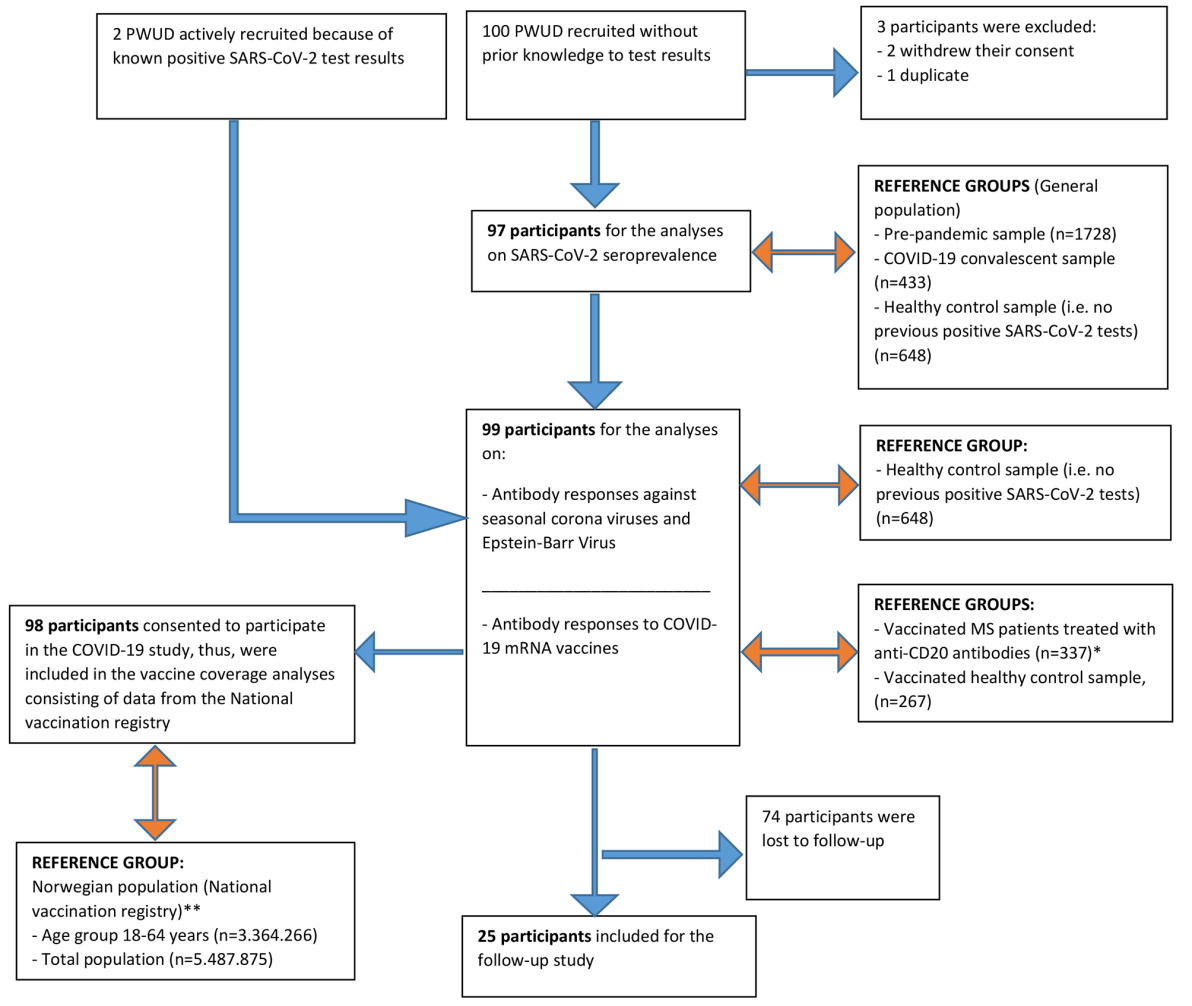
Work flow chart of participants and reference groups. *Note*. *) Based on published data from a cohort of vaccinated patients treated with anti-CD20 antibodies for Multiple Sclerosis [29] **) Data first published in the weekly report no 52/2022 by the Norwegian Institute of Public Health [30]

See work flow chart for details on study populations and reference groups (Fig. 1).

### Data collection

*The first data collection* was conducted from November 19th 2020 to February 9th 2021, before the vaccines against SARS-CoV-2 became available in Norway. It was based on a questionnaire that included information about age, sex, use of drugs, years of injecting drug use, status of vaccination (hepatitis A and B and the seasonal flu), antiviral treatment (hepatitis C and HIV), former SARS CoV-2 PCR test results, symptoms of covid-19 and other different chronic diseases. “Years of injecting drug use” was defined as the number of years with injecting drug use, independent of the number of injections per year. Relevant health information was collected from patient records. Swabs from nasopharynx and throat were collected and sent to the Department of Microbiology at Oslo University Hospital for SARS CoV-2 PCR test [31]. Blood samples were collected to test for antibodies against SARS-CoV-2 and other viruses like seasonal coronaviruses and EBV (see below). We included analyses for antibody responses against seasonal corona viruses and EBV because the seroprevalence for these viruses is close to 100% in the adult population. We included results for antibodies to EBV, since responses to this virus was not expected to be influenced by the lockdown.

*The second data collection* was to be conducted within 3 months after the participants had received their second dose of vaccine against SARS-CoV-2 and took place in November 2021. Twenty five participants from the original cohort were interviewed with a shortened version of the baseline questionnaire, and blood samples were collected and tested for antibodies as described above.

*Data on vaccination* against SARS-CoV-2 was collected from the National Vaccine Register (SYSVAK) until the end of 2022. We chose this end date to cover a period when most people had received the recommended number of vaccine doses against SARS-CoV-2.

The participants received 100 Norwegian krone (approximately 10 euros) for participation in the study at baseline. At the second round of participation, the participants received 200 Norwegian krone (about 20 euros).

### Serologic methods

**Fig. 2:**
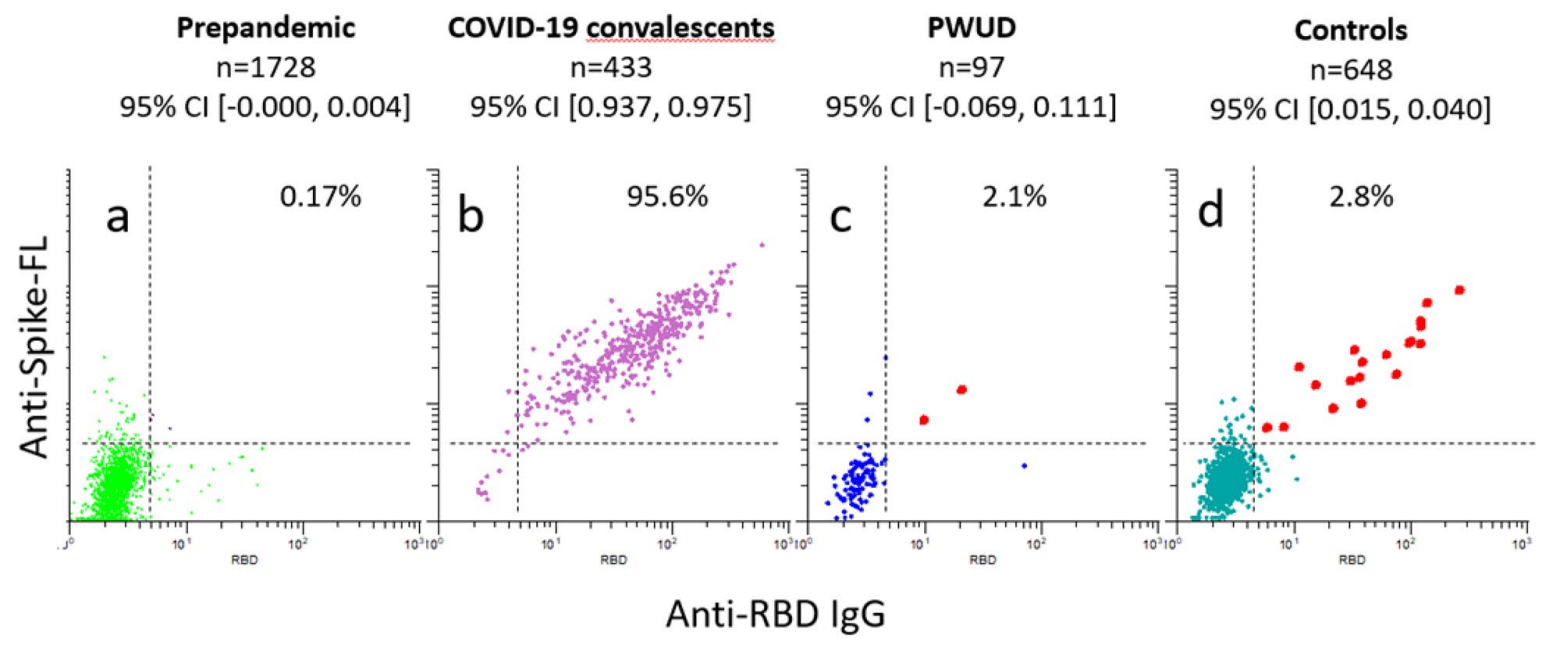
Measurements of SARS-CoV-2 seroprevalence. *Note*. The dot plots show levels of antibodies (log 10) to indicated SARS-CoV-2 proteins in sera from indicated cohorts. Each dot represents a different sample. Spike-FL: full-length spike protein from SARS-CoV-2. RBD : receptor-binding domain. Bead-based arrays with virus proteins were incubated with serum diluted 1:100, labelled with R-Phycoerythrin (R-PE)-conjugated anti-human IgG Fc and analyzed by flow cytometry. The values correspond to R-PE median fluorescence intensity (MFI) measured for beads with indicated virus protein divided by the MFI measured for beads with neutravidin only. The dashed lines correspond to cutoffs set by analyzing prepandemic samples (a)

Antibodies to SARS-CoV-2, seasonal coronaviruses and Epstein-Barr virus were measured using a bead-based flow cytometric assay as described in detail earlier [32, 33]. Since the seroprevalence in Norway was lower than 2% for most of 2020, we used a double cut-off to yield fewer than 0.2% positives in pre-pandemic sera [34]. This implies that samples had to be positive for antibodies against the full-length Spike protein from SARS-CoV-2 as well as to the receptor-binding domain (RBD) (Fig. 2) A similar strategy has been reported by others [35].

### Data analysis

Numbers are presented as percentages (%) and frequencies (*n*). SPSS version 28.0 was used for statistical analyses [36].

Flow cytometry data were analysed in WinList 3D. Median fluorescence intensity (MFI) of measured from beads with virus proteins was divided by that measured from beads with neutravidin only to determine relative MFI (rMFI).

To assess the seroprevalence among PWUD, we measured antibodies to Spike-FL, and RBD from SARS-CoV-2 in sera obtained in November 2020. Results obtained with pre-pandemic sera and sera from individuals with PCR-confirmed COVID-19 convalescents showed that a double cut-off for anti-RBD and anti-spike yielded a sensitivity of 98.2% and a specificity of 0.3%. (Fig. 2a-b).

For the purpose of sensitivity analyses, we also analysed the seroprevalence of SARS-CoV-2 including the two participants that were actively recruited because of known positive SARS-CoV-2 PCR test results.

To assess antibody responses to vaccination, we used a serially diluted serum as standard to convert rMFI to binding antibody units per millilitre (BAU/ml) for standardized measurement of antibodies to RBD from SARS-CoV-2 [32].

RMFI values measured for antibodies to seasonal coronaviruses and EBV were compared to those measured in healthy individuals. Significance of difference was analysed using the Mann-Whitney non-parametric test.

## Results

### Questionnaires

#### Participants

At baseline, questionnaires, swabs and blood samples were collected from 102 participants, of which two people withdrew their consent, and one duplicate was found (one person participated twice). This gives a total of 99 participants at baseline, with 97 participants being eligible for the seroprevalence analyses Ninety-eight participants gave additional consent to take part in the larger COVID-19 study. (See Fig. 1).

Of the participants at baseline, 76.8% were male and the mean age was 44 (range 22–67). 25 persons participated in the second data-collection. Of them, 76.0%) were male and the mean age was 46 (range 27–60) (see Table 1).

**Table 1:** Baseline and follow-up questionnaires

QUESTIONNAIRE	BASELINE (*N* = 99)			
**Type of drugs usually taken**	***n***		**Valid per cent**	
Heroin	72		72.7	
Amphetamine	53		53.5	
Benzodiazepines	29		29.2	
Cannabis/THC	44		44.4	
Alcohol	10		10.1	
Cocaine	4		4.0	
GHB	5		5.1	
Other	4		4.0	
**Drug administration route**	***n***		**Valid per cent**	
Oral	30		30.3	
Smoking or nasal	89		89.9	
Injection	68		68.7	
Several administration routes including injection	26		26.3	
**Vaccination (yes)**	***n***		**Valid per cent**	
Hepatitis A and B (*N* = 85)	35		41.2	
Seasonal flu (*N* = 87)	14		16.1	
**QUESTIONNAIRE**	**BASELINE (** ***N*** ** = 99)**		**FOLLOW-UP (** ***N*** ** = 25)**	
**Sociodemographics**	**Mean**	**SD**	**Mean**	**SD**
Age	44	11.2	46	10.4
	***n***	**%**	***n***	**%**
Sex (male)	76	76.8	19	76.0
**Antiviral treatment (yes)**	***n*** **(** ***N*** **)**	**Valid %**	***n*** **(** ***N*** **)**	**Valid %**
Hepatitis C (previous or current)	27 (87)	31.0	2^a^ (25)	8.0
HIV (ongoing)	2 (92)	2.2	1 (25)	4.0
**Tested for SARS-CoV-2-RNA (PCR)**	***n*** **(** ***N*** **)**	**Valid %**	***n*** **(** ***N*** **)**	**Valid %**
Yes (once or several times)	53 (99)	53,5	21 (25)	84,0
Don’t remember	1 (99)	1.0	-	-
**Symptoms at the time of PCR-test**	***N*** = 53		***N*** = 21	
	***n*** = 43	**Valid %**	***n*** = 20	**Valid %**
None (routine-testing)	36	83.7	10	50.0
Yes	7	16,3	3	15.0
Don’t remember^b^	-	-	7	35.0
**Test results from previous PCR-tests**	***N*** = 53		***N*** = 21	
	***n*** = 53	**Valid %**	***n*** = 21	**Valid %**
Positive	2^c^	3.8	2	9.5

*N* is the number of eligible participants answering the questions, *n* is the number of participants answering to the item. Valid percentages are given

^a)^ 1 person had ongoing HCV-treatment at the time of the questionnaire and blood test

^b)^ This option was only in the follow-up questionnaire

^c)^ Both patients were recruited because of the known positive test result for SARS-CoV-2

#### Drug use and vaccination status at baseline

Heroin, amphetamine, and cannabis were the drugs most usually taken, and smoking/nasal or injection were the most common routes of drug administration (see Table 1). Eighty participants answered the question about how many years they had injected drugs. The mean number of years of injecting drug use was 16.7 (range 0–50, SD: 13.3). Eleven participants (13.8 valid %) had never injected (data not shown in table).

Regarding vaccination status concerning hepatitis A and B and the seasonal flu: About 41% had received vaccination against hepatitis A and B and 16% had received vaccination against the seasonal flu (Table 1). Eighteen (21.2 valid %) said they did not know whether they had received a vaccination against hepatitis A and B (data not shown in table).

#### Antiviral treatment and previous testing for SARS-CoV-2 at baseline and follow-up

The questions about antiviral treatment and previous testing for SARS-CoV-2 were asked both at baseline and follow-up. About one third of the baseline participants had undergone treatment for hepatitis C (Table 1). Amongst the 25 participants from the second data collection, two had received hepatitis C treatment since the baseline questionnaire, one of them was on ongoing treatment. Two of the baseline participants answered that they were currently on antiviral treatment for HIV. One of them also participated in the second data-collection (Table 1).

In the first questionnaire, about half the participants answered that they had been tested for SARS-CoV-2 at least once. About 2/3 of the tested participants had undergone routine testing (i.e. related to hospital admission or quarantine) with no symptoms at the time, and two had received a positive result for SARS-CoV-2. Both of the participants that had a previous positive test were actively recruited because of the known positive results. Between the first and second data collection, 84 per cent of the participants at follow-up had been tested at least once, most of them had undergone routine testing (i.e. without symptoms) and 2 reported that they had a positive test since the first data collection. (See Table 1).

### Results from SARS-CoV-2 PCR and antibody tests

#### SARS-CoV-2 PCR swab test

During the first data collection, one out of the 99 swab samples was positive for SARS-CoV-2 by PCR. This sample was collected from the participant that was recruited from the institution for isolation due to an ongoing SARS-CoV-2 infection (data not shown in table).

#### The seroprevalence of SARS-CoV-2 in people who use drugs in 2020 compared to that in the general population

**Fig. 3:**
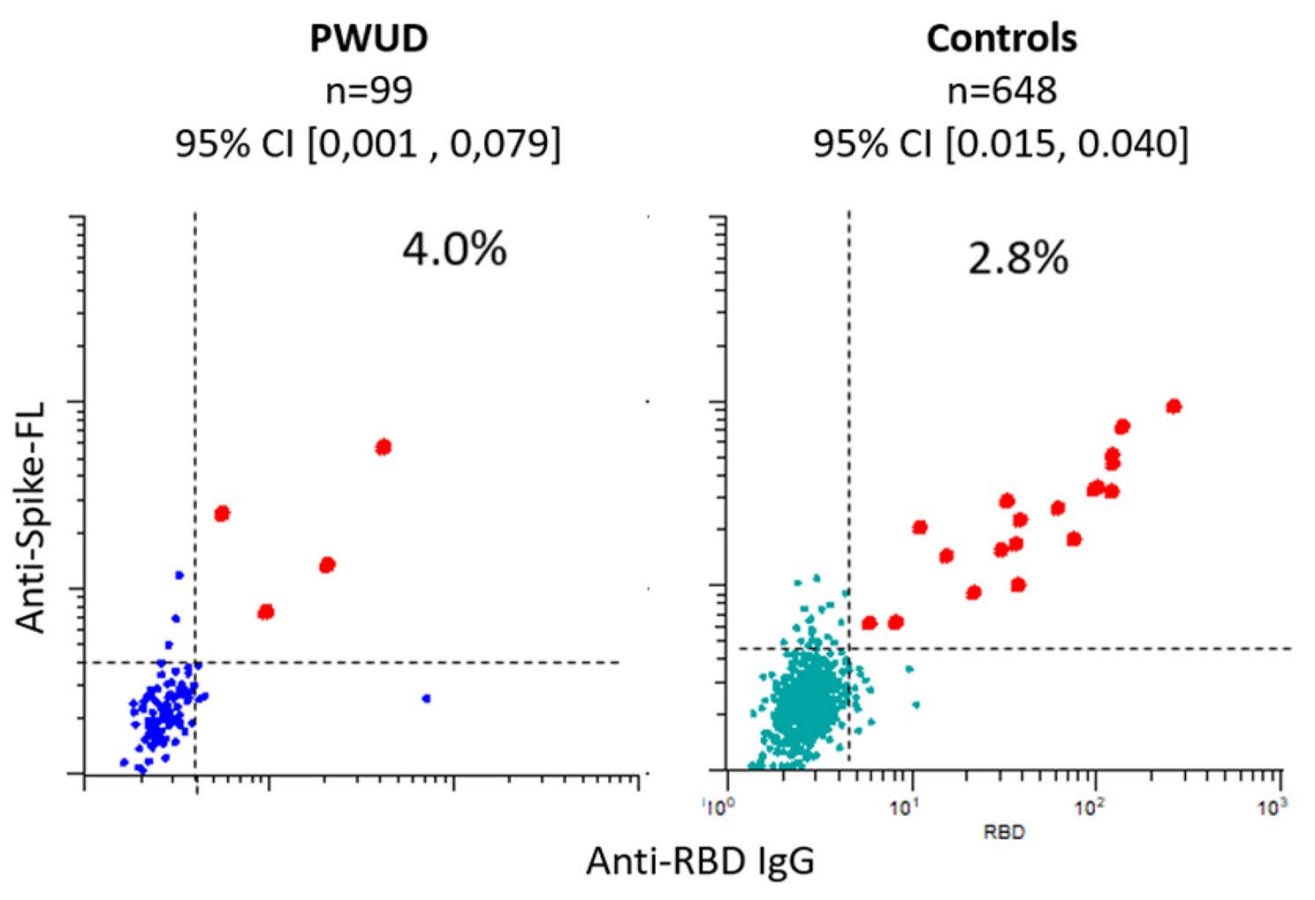
Sensitivity analyses of SARS-CoV-2 seroprevalence. *Note*. The dot plots show levels of antibodies (log 10) to indicated SARS-CoV-2 proteins in sera from indicated cohorts. Each dot represents a different sample. Spike-FL: full-length spike protein from SARS-CoV-2. RBD : receptor-binding domain. Bead-based arrays with virus proteins were incubated with serum diluted 1:100, labelled with R-Phycoerythrin (R-PE)-conjugated anti-human IgG Fc and analyzed by flow cytometry. The values correspond to R-PE median fluorescence intensity (MFI) measured for beads with indicated virus protein divided by the MFI measured for beads with neutravidin only. The dashed lines correspond to cutoffs set by analyzing prepandemic samples (Fig. 2a)

Two out of 97 (2.1%) samples from PWUD were above the cut-off specificity (99.5%) and sensitivity (92%) (95% CI [-0.07, 0.11]) (Fig. 2c). The corresponding frequency for population-based screening in 2020 was 2.8% (95% CI [0.02, 0.04]) (Fig. 2d). Sensitivity analyses including all the participants in the baseline cohort (*N* = 99) gave a prevalence estimate of 4.0% (95% CI [0.00, 0,08]) (See Fig. 3). Since the 95% Confidence Intervals (CI) of the prevalence estimates overlap between the study population, the control sample, and the sensitivity analyses, we conclude that the seroprevalence of SARS-CoV-2 in the study population is similar to that of the general population.

#### The levels of serum antibodies to seasonal coronaviruses and EBV among people who use drugs compared to those observed in the general population

**Fig. 4:**
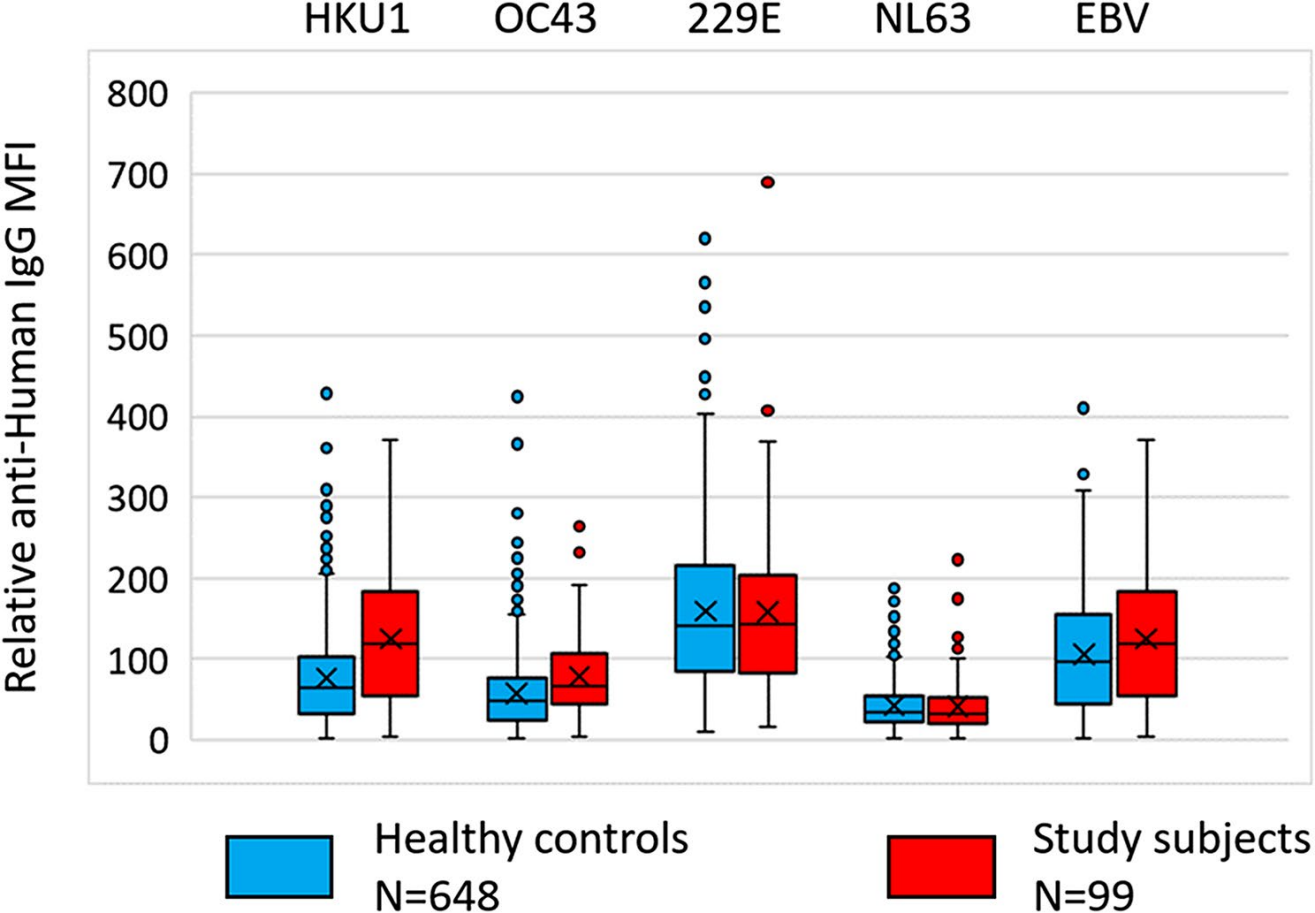
Levels of antibodies to seasonal coronaviruses and EBV in people who use drugs (PWUD) and controls. *Note*. The bar and whisker plot shows levels of antibodies to spike proteins (S1 domains) from indicated virus and the EBNA1 protein from EBV in PWUD (*n* = 99) and healthy controls (*n* = 648). The values correspond to anti-IgG R-PE median fluorescence intensity (MFI) measured for beads with indicated virus protein divided by the MFI measured for beads with neutravidin only

The bead-based arrays also measured spike proteins (S1 domains) from seasonal coronaviruses. We also included results for antibodies to nuclear protein EBNA1 from EBV. Since the sera were analysed at a single dilution (1:100), we cannot estimate exact titres. *However, the results show that the levels measured in PWUD were at least as high as those measured in sera obtained from individuals included in population-based screening. Thus, we neither found any evidence of immunodeficiency among PWUD nor any pre-existing increased immunity* (Fig. 4).

### Vaccination rates and responses to COVID-19 mRNA vaccines

At the time of the second data collection (November 2021), all 25 participants had received dose 1 and 24 had received dose 2 of the vaccines against SARS-CoV-2. The one participant that had received only one dose had a confirmed positive SARS-CoV-2 PCR test 46 days before the second data collection. Another participant had a positive SARS-CoV-2 PCR test 10 days prior to the assessment. The blood samples for the second data collection were to be taken within 3 months after the second dose of vaccination, however, the mean number of days between dose 2 (or confirmed case of COVID-19) and the data collection was 137 days (range 10–208, SD: 52.1) (data not shown in table).

By the end of 2022, 84% of the study participants had received the first dose of vaccination, 74% had received dose 2, 31% had received dose 3 and 3% had received dose 4. In the general population, the corresponding percentages from the equivalent age group (18–64 years) were 89%, 86%, 60%, and 5%, respectively (see Table 2).

**Table 2:** Vaccines against SARS-CoV-2 given from the time they became available and through 2022. The study population of people who use drugs (PWUD) compared to the general population

	PWUD			General population*	
	*N* = 98			Age group 18–64 years *N* = 3 364 266	Total population *N* = 5 487 875
	*n* (%)	95% CI		%	%
		Lower	Upper		
Dose 1	83 (84)	0.77	0.91	89%	77%
Dose 2	73 (74)	0.65	0.83	86%	72%
Dose 3	31 (31)	0.22	0.40	60%	53%
Dose 4	3 (3)	-0.00	0.06	5%	16%

Study participants all received COVID-19 mRNA vaccines

*N* = number of population and study participants

*n* = number of study participants receiving vaccine against SARS-CoV-2

% = per cent

95% CI = 95% confidence interval. Considering the very large sample size of the general population samples, giving a very narrow 95% CI, only the point estimates are provided for these prevalences

* Data first published in the weekly report no 52/2022 by the Norwegian Institute of Public Health [30]

### Responses to COVID-19 mRNA vaccines in people who use drugs compared to those observed in healthy individuals and individuals on immunosuppressive treatment

**Fig. 5:**
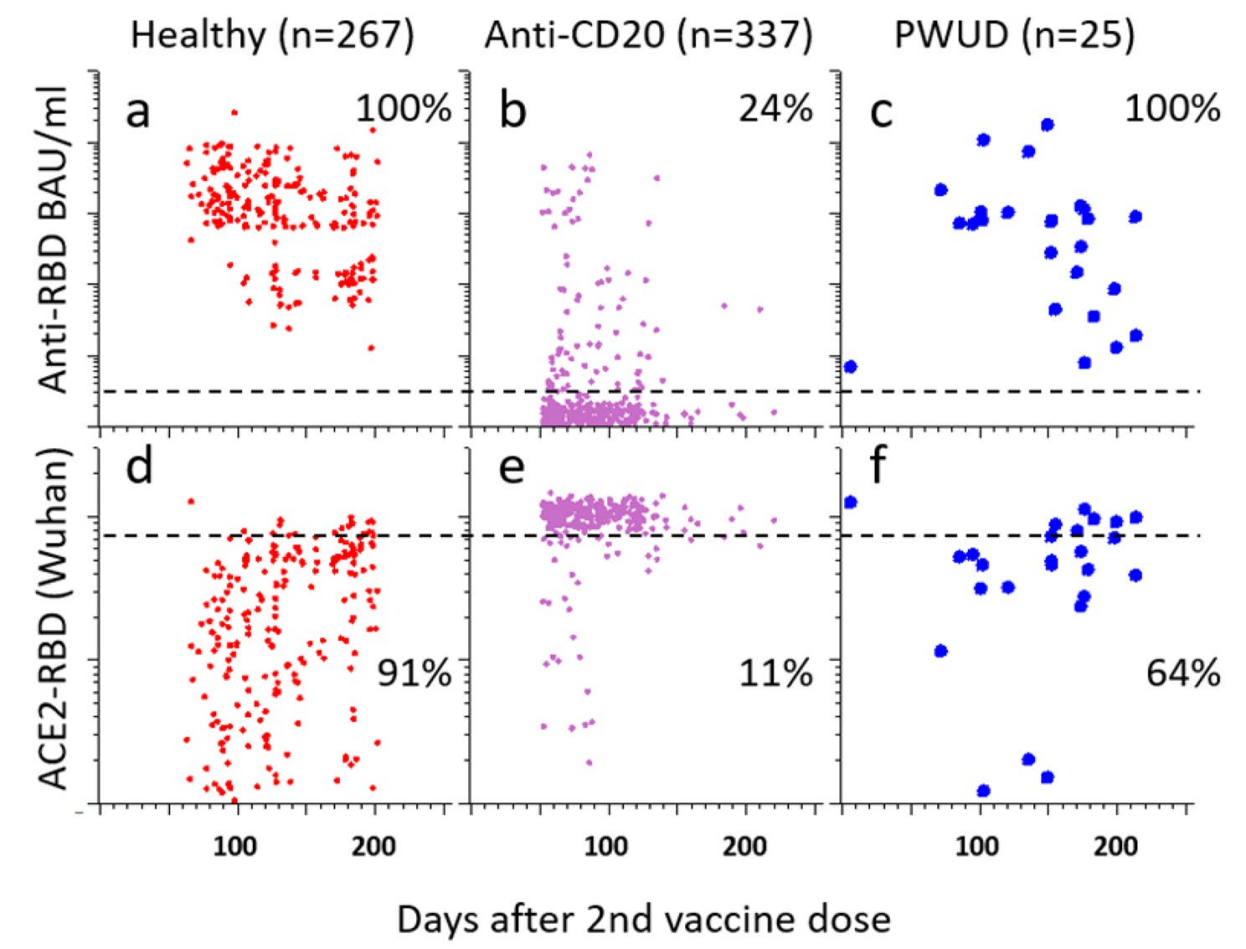
Levels of antibodies to the receptor-binding domain (RBD) of the SARS-CoV-2 spike protein in sera obtained after COVID-19 vaccination of people who use drugs (PWUD), people treated with anti-CD antibodies for Multiple Sclerosis and healthy controls. *Note*. **a-c**) The dot plots show levels of antibodies to the receptor-binding domain (RBD) of SARS-CoV-2 measured in sera obtained at indicated time point (x-axis) after the 2nd COVID-19 mRNA vaccine dose. Each dot corresponds to a different sample. Sera were obtained from healthy individuals (*n* = 267), MS patients on anti-CD20 therapy (*n* = 337)*) and PWUD (*n* = 25). Signal values were converted to binding units per millilitre (BAU/ml) as described in the methods section. The dashed line corresponds to the detection limit. **d-f**: The dot plots show inhibitory effects of the sera on binding of ACE2 to RBD (y-axis) versus days after the 2nd COVID-19 mRNA vaccine dose. Values below the dashed line are considered to indicate neutralizing activity. *) Based on published data from a cohort of vaccinated patients treated with anti-CD20 antibodies for Multiple Sclerosis [29]

To assess humoral immune responses to SARS-CoV-2 Spike mRNA vaccination, we measured binding- and neutralizing antibodies to the RBD from SARS-CoV-2 in sera obtained from the 25 participants described above. Results obtained with samples from healthy individuals or patients with multiple sclerosis who were treated with B-cell-depleting anti-CD20 antibodies were included as positive and negative controls, respectively [29] (Fig. 5a-b, d-e). Antibodies were barely detectable in the sample obtained 8 months after a single COVID-19 mRNA vaccine and in two samples from PWUD obtained six months after the 2nd dose (Fig. 5c). Most samples from PWUD were obtained more than 115 days after the second dose. The median anti-spike titer at this time was 445 Binding Antibody Units per milliliter (BAU/ml), compared to 739 BAU/ml in samples obtained from healthy controls 115 days after the second dose (*p* = 0.07, ns) (Fig. 5a, c*).*

The results in Fig. 5d-f show time-dependent variation in the inhibitory effects of sera on ACE2-binding to RBD from SARS-CoV-2 (Wuhan). Low ACE2-binding indicates high levels of neutralizing antibodies, and the signals measured in most samples from MS patients on anti-CD20 therapy serve as an example of a total lack of neutralizing activity (Fig. 5e). Inhibition of ACE2-binding to RBD was observed in 91% of sera from healthy controls. The corresponding frequency in PWUD was 64%, but it is worth noting that most sera were obtained later than 150 days after vaccination. The median inhibition of ACE2-binding to RBD was 56% and 66% in samples from PWUD and healthy controls, respectively (*p* = 0.1, ns) (Fig. 5d, f). *While the number of samples from PWUD was small, we conclude that there was no clear difference between PWUD and healthy individuals with regard to humoral immune responses to COVID-19 vaccination.*

## Discussion

We conducted this study as a response to the surprisingly low number of SARS-CoV-2 PCR confirmed cases in this group during the first wave of the pandemic in Oslo. We postulated that people in this group could have had asymptomatic infections and therefore had not been tested, that there had been little contact between the general population and this group and thus a low spread of the virus into the group, or that they had acquired protective immunity towards SARS-CoV-2 through previous virus infections, like the seasonal coronavirus. However, we found the seroprevalence of SARS-CoV-2, the serologic responses to seasonal coronaviruses and EBV, the humoral immune responses to COVID-19 mRNA vaccines, and the vaccination coverage to be similar between PWUD and the general population.

The participants of this study were mostly male with a mean age of 44 years. The preferred drugs of use were heroin, amphetamines and cannabis and the preferred routes of administration were smoking/oral and injection. This is comparable to other studies from this group [28, 38, 39].

In our study, we found that about half the participants at baseline had a previous PCR-test of SARS-CoV-2 and that most of them had undergone routine testing (i.e. without symptoms). None of the asymptomatic cases had received a positive PCR-test result. We found that the seroprevalence of SARS-CoV-2 among our participants was comparable to that in the general population. There are only a few other studies on seroprevalence of SARS-CoV-2 in people with substance use disorders (SUD) compared to the general population. Djuric and colleagues conducted a registry-based study with data from patients in addiction services and the general population in Northern Italy. They found a lower risk of having SARS-CoV-2 among people with SUD compared to the general population even though they had a higher risk of being tested for SARS-CoV-2 [40]. Similarly, a study by Lindquist and colleagues found a lower prevalence of SARS-CoV-2 among people with SUD attending a needle exchange program in Stockholm [41] when comparing to the prevalence of SARS-CoV-2 found in a study on healthy blood donors from the same region and period [42]. A study by Vallecillo and colleagues, found a similar incidence of COVID-19 among patients on OAT [43] compared to an age-reference general population [44].

These studies together with ours, point in another direction than the many studies finding a higher prevalence of SARS-CoV-2, more severe morbidity and greater mortality related to the disease among PWUD [22–25]. Strathdee and colleagues found a prevalence of SARS-CoV-2 among PWUD in the San Diego-Tijuana border to be higher than estimates from the general population in either city [45, 46]. Wang and colleagues did a retrospective case-control study of electronic health records. They found a significantly increased risk of COVID-19 among patients with a recent diagnosis of substance use disorder [22]. Vai and colleagues did a systematic review and meta-analysis from studies reporting data on COVID-19 outcomes among patients with mental disorders compared to controls without mental disorders. They found that the presence of any mental disorder (including substance use disorders) was associated with an increased risk of COVID-19 related mortality, with an even higher odds ratio for mortality in individuals that were not admitted to hospital for their SARS-CoV-2 infection. Having a comorbid substance use disorder was also associated with an increased risk of hospitalization after SARS-CoV-2 infection [23].

Our findings are also in contrast to a nationwide seroprevalence study in Denmark among people experiencing homelessness (PEH) and staff by Eriksen and colleagues [47]. They found that the seroprevalence among both people living in the shelters and shelter workers were more than twice as high as that of the general population. In line with these findings, another study from Denmark and a study from the USA found higher seroprevalence in geographical areas with lower socioeconomic status [48, 49]. Bagget and colleagues, describe a rapid increase in COVID-19 cases in a large shelter for PEH in Boston, where almost 90% of positive cases reported no symptoms [21]. This is an example of a scenario we feared and aimed to prevent by establishing extensive mitigation strategies. Karb and colleagues found that SARS-CoV-2 prevalence varied with shelter characteristics like resident stability and physical distancing [50]. These findings are supported by several studies showing higher seroprevalence of SARS-CoV-2 among people living in unstable housing like emergency shelters [51, 52], and that risk factors for seropositivity were most strongly associated with crowded living conditions and sharing a bathroom with more than five people [53].

During the first wave of the pandemic, personal protection equipment, like masks, were scarce and only to be used by health personnel treating vulnerable patients. Thus, the main pillar for infection control was social distancing together with reducing mobility and optimizing stability among both residents and staff. Questions have been raised whether marginalized people like PEH and PWUD would be willing and able to comply with infection control strategies. Welle-Strand and colleagues conducted two surveys among PWUD and found that many of the respondents were positive to be tested for SARS-CoV-2 and to comply with National infection control recommendations [54, 55]. In contrast, a study by Lindqvist and collegues found that even though the vast majority of PWUD had correct knowledge about transmission routes, protective measures and personal risk factors, 38% of participants with a suspected or confirmed SARS-CoV-2 infection reported that they did not change their behaviour during illness [41]. The participants in our study, were living in low-threshold, temporary municipal housing facilities for people with severe drug use, where it was hard to comply with National infection control guidelines as most of these housing facilities have shared bathrooms and kitchens. The residents needed to have close contact for several reasons, and even though we had made recommendations for staff to keep distance to residents and colleagues, this was hard to comply with during the course of the day. Because of these circumstances, and in line with the above-mentioned studies, the risk of spread of the virus could potentially be high if one or more residents or staff were to be infected.

One of our prior hypotheses was that PWUD had acquired protective immunity towards SARS-CoV-2 through previous repeated exposure and infections with one or more of the seasonal coronaviruses. This hypothesis was in contrast to the many risk evaluations stating that PWUD would have reduced immunity towards SARS-CoV-2 due to the use of opioids [56] and chronic infections like HIV and viral hepatitis and also liver cancers [57]. However, we found that the serological immunity towards seasonal coronaviruses among PWUD were similar to the general population. Similarly, the levels of antibodies towards EBV was not different from the general population, as expected, as this long-lasting immunity is generally acquired during childhood and adolescence [58].

As none of our predictions regarding the prevalence of infected and severely ill people in this group were met, one might ask if the extensive tailored mitigating strategies we employed were wasted efforts and resources. However, in a systematic review including 37 studies, Mohsenpour and colleagues found that a baseline SARS-CoV-2 infection prevalence of 2.3% among PEH in homeless shelters would increase drastically to 31.6% in case of an outbreak situation [59]. Similarly, a modelling study by Lewer and colleagues found that outbreaks of SARS-CoV-2 in homeless settings could lead to a high infection rate among PEH in spite of a low incidence in the general population [60]. Thus, the seroprevalence was kept low among PWUD in Oslo as a result of a combination of the general, National mitigating strategies, keeping the seroprevalence low in the general population, and the tailored strategies, keeping the potential spread low in case of a local outbreak in the institutions for PWUD. A study from Ireland by O’Carrol and colleagues, describes similar tailored mitigating interventions to those that we employed. They also experienced surprisingly low morbidity and mortality of COVID-19 among homeless PWUD. They concluded, that this could be the result of a well-coordinated government policy that was underpinned by a science driven and fundamentally pragmatic approach. They argued that the rapid collapse in policy barriers during this crisis made these interventions possible and that they should be secured and protected in the provision of services to this group in the future [61].

That said, these tailored approaches are also extremely resource demanding on several levels; on the service level, on the staff, and on the users of these services. Complying with infection control and mitigating measures, including quarantine and isolation, is especially demanding for the most vulnerable individuals; those with chaotic lives and reduced functioning in several areas of life, and that are also most at risk for severe courses of COVID-19. Many have therefore advocated that PWUD should be amongst the first groups to be prioritized for the vaccine against SARS-CoV-2 as soon as it became available [62, 63]. In Norway, PWUD were included among the prioritized groups for vaccination right after elderly and people with chronic diseases with vaccines becoming available for this group from April 2021.

We collected data on vaccination until the end of 2022. The numbers show similar percentages for receiving dose 1 and dose 4 between the study population and the corresponding age group of the general population. Vaccine coverage is declining with dose numbers in both groups, although they seem to be declining more among PWUD. However, the percentage of PWUD who received dose 2 is still on level with the total Norwegian population [30]. In contrast, several studies have found a lower cumulative vaccine uptake mong PWUD compared to the general population [64–69]. Some studies suggest that this can be explained by hesitancy towards the vaccines among PWUD [65, 67]. Cepeda and colleagues found that PWID who somewhat trusted or did not trust vaccination had 6 times higher odds of being unvaccinated [65]. Other studies highlight the importance of lowering the barriers to receive vaccination, like building trust and tailoring services to this group [63, 70, 71]. Welle-Strand and colleagues conducted a survey among PWUD during the first three months of 2021. They found that 58% of the responders had positive attitudes towards vaccination against SARS-CoV-2 [55]. The vaccination for marginalized PWUD in the present study was provided through already established and trusted low-threshold health services for this group, which probably lowered the barriers to receive the vaccines.

Another unanswered question was to which degree PWUD would gain from the vaccination against SARS-CoV-2. Although the humoral immune responses have been extensively studied in general [72], and among people with different chronic diseases [37, 73, 74], there have not been any studies investigating the humoral immune responses against SARS-CoV-2 in PWUD. We compared the humoral immune responses towards SARS-CoV-2 in PWUD to patients with Multiple Sclerosis on anti-CD-20 therapy and to healthy controls. We found that the humoral immune responses to COVID-19 mRNA vaccines from PWUD was comparable to levels observed in healthy controls. This finding underscores the importance and effectiveness of vaccinating PWUD against SARS-CoV-2.

### Strengths and limitations

There are several limitations of this study. Firstly, the sample is small, which might lead to underestimation of differences between the study population and referral groups (type 2 statistical error). Further, two of the participants at baseline were recruited because of known positive SARS-CoV-2 PCR test results. However, sensitivity analyses with and without these samples yielded similar results with overlapping 95% confidence intervals. Further, this is a clinical study that was conducted during the pandemic and with the aid of clinicians that, in spite of being extremely dedicated to this research, had to prioritize clinical tasks such as testing and vaccinating this vulnerable group of patients. We were therefore only able to include a smaller part of the original baseline cohort at follow-up.

## Conclusions

Results showed that PWUD did not have increased serological responses to seasonal coronavirus and therefore no evidence of increased pre-existing immunity. The low prevalence of positive SARS-CoV-2 cases among PWUD was probably the result of a combination of the comprehensive general and the tailored mitigating strategies that were employed. Vaccine responses and coverage were not different from controls demonstrating that vaccination is a viable strategy to confer protection against SARS-CoV-2 in PWUD.

## Data Availability

The datasets used and/or analysed during the current study are available from the corresponding author on reasonable request.
